# Group A streptococcal septicaemia presenting as an acute abdomen in a child

**DOI:** 10.1186/1749-7922-2-15

**Published:** 2007-06-05

**Authors:** Melissa Short, Anne Lawson

**Affiliations:** 1 Department of Paediatric Surgery, Royal Victoria Infirmary, Newcastle upon Tyne, England, UK

## Abstract

We present an unusual case of group A streptococcal septicaemia referred to a paediatric surgical unit as acute appendicitis and highlight the importance of remembering this condition as part of a differential diagnosis.

## Case report

Miss AL, a six year old girl was seen in A&E with pyrexia, diarrhoea and vomiting, hip and shoulder pain pyrexia of two days duration. She had also complained of a sore throat and earache of four days duration. Her symptoms were felt to be consistent with a viral upper respiratory tract infection and gastroenteritis. No investigations were performed at this time and she was discharged with Diarolyte.

The patient returned four days later with persistent multi-joint pains; having now developed diffuse abdominal pain and distension and at this point a surgical opinion was sought.

The child was pyrexial and hypoxic, and clearly unwell. Ear, nose and throat examination was normal at this time as it had been one week previously. She had peritonitis localised to the right iliac fossa and an overlying cellulitis. Note was also made of an intermittent roving erythematous but non pruritic rash over her wrists and distal limbs.

The full blood count (white cell count 25.6, neutrophils 23.1 platelets 47) and C-reactive protein (248 mg/L) were consistent with fulminant sepsis.

Following an equivocal abdominal ultrasound scan the patient was taken to theatre.

Laparotomy revealed copious fibrin deposits and a congested caecum and appendix which were not felt to be due to acute appendicitis. appendicectomy was performed.

Despite aggressive fluid resuscitation and broad-spectrum antibiotics (cefuroxime, metronidazole and gentamicin) the patient deteriorated rapidly, developing disseminated intravascular coagulation, acute respiratory distress syndrome and acute renal failure. She was kept intubated and required admission to the paediatric intensive care unit for inotropic and ventilatory support for 48 hours post operatively. Blood cultures from admission and peritoneal swabs from laparotomy grew a fully sensitive group A streptococcus at 48 hours, after which her antibiotics were rationalised and she was commenced on clindamycin. Her condition improved and she was discharged from hospital after two weeks with no post infection sequelae to date.

## Discussion

Group A streptococcus is one of the most frequent pathogens in humans, a normal upper respiratory tract commensal in 5–15% of normal individuals [1], responsible for a broad spectrum of clinical entities, the most commonly occurring illnesses being streptococcal tonsillitis, scarlet fever and more rarely, streptococcal toxic shock syndrome (STSS) [2]. First described at the end of the last century, STSS (Group A streptococcal infection associated with the early onset of shock and organ failure. [3]) carries a significant morbidity and mortality (up to 70% [4]), is more common in adults than children and is increasingly being reported in patients who have no predisposing factors [5]. Over the last 15 years however, there has been a significant increase in the incidence and severity of infections caused by Group A Strep, including STSS seen in children [6], thought to be a response to more virulent and resistant strains of the bacteria evolving. It is more likely to mimic peritonitis than appendicitis, and is therefore an essential differential to be borne in mind when confronted with a peritonitic, rapidly deteriorating previously well child following an initial upper respiratory tract infection, which has failed to improve despite simple medical management.

There are very few case reports of children with Group Strep A. presenting as Appendicitis. We have come across only 3 – 4 case reports in English literature from 1965. Hence we would like to re-emphasis the need to keep this medical condition on our differential diagnosis while treating appendicitis.
